# Supplementary material for: Characterization of Matrix Metalloprotease-9 Gene from Nile tilapia (Oreochromis niloticus) and Its High-Level Expression Induced by the Streptococcus agalactiae Challenge
Source: Biomolecules. 2020 Jan 3;10(1):76. doi: 10.3390/biom10010076 (PMC7023376; doi:10.3390/biom10010076)
Supplement: Supplementary file 1 [file biomolecules-10-00076-s001.pdf]

# Supplementary Information

**Journal name:** ***Biomolecules***

**Title: Characterization of matrix metalloprotease-9 gene from Nile tilapia (*Oreochromis niloticus*) and its high-level expression induced by *Streptococcus agalactiae* challenge**

**Author's names: Fu-Rui Liang[1†], Qin-Qing Wang[1,2†*], Yun-Lin Jiang[1], Bei-Ying Yue[1], Qian-Zhi Zhou[1], Jiang-Hai Wang[1,2,3*]**

**Affiliations:**

[1]Guangdong Provincial Key Laboratory of Marine Resources and Coastal Engineering, School of Marine Sciences, Sun Yat-Sen University, Guangzhou 510006, China

[2]Southern Marine Science and Engineering Guangdong Laboratory (Zhuhai), Zhuhai 519000, China

[3]South China Sea Bioresource Exploitation and Utilization Collaborative Innovation Center, School of Marine Sciences, Sun Yat-Sen University, Guangzhou 510006, China

[†]The authors contributed equally to this work.

* Corresponding authors: wangjhai@mail.sysu.edu.cn (J.-H. Wang);
wangqq39@mail.sysu.edu.cn (Q.-Q. Wang).

Tel.: +86 20 39332212.

# Supplementary figures

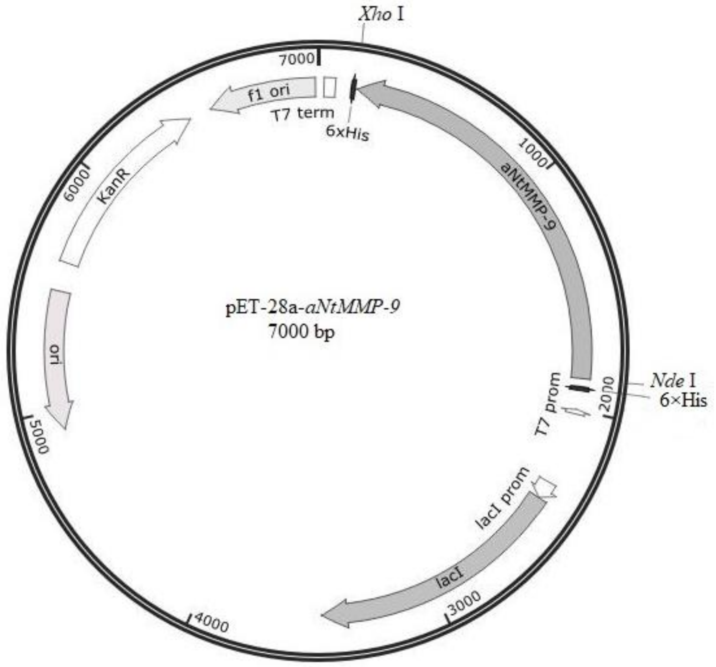


**Figure S1.** Reconstruction of the expression vector pET-28a/*aNtMMP-9*

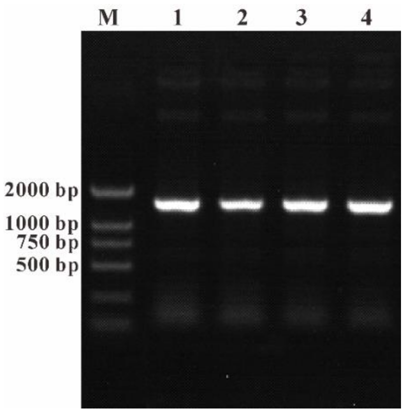


**Figure S2.** Identification of positive transformants containing pET-28a/*aNtMMP-9* vector amplified by PCR.

M, DNA marker DL2000; Lanes 1-4, the bands (1,716 bp) were amplified from different transformants.

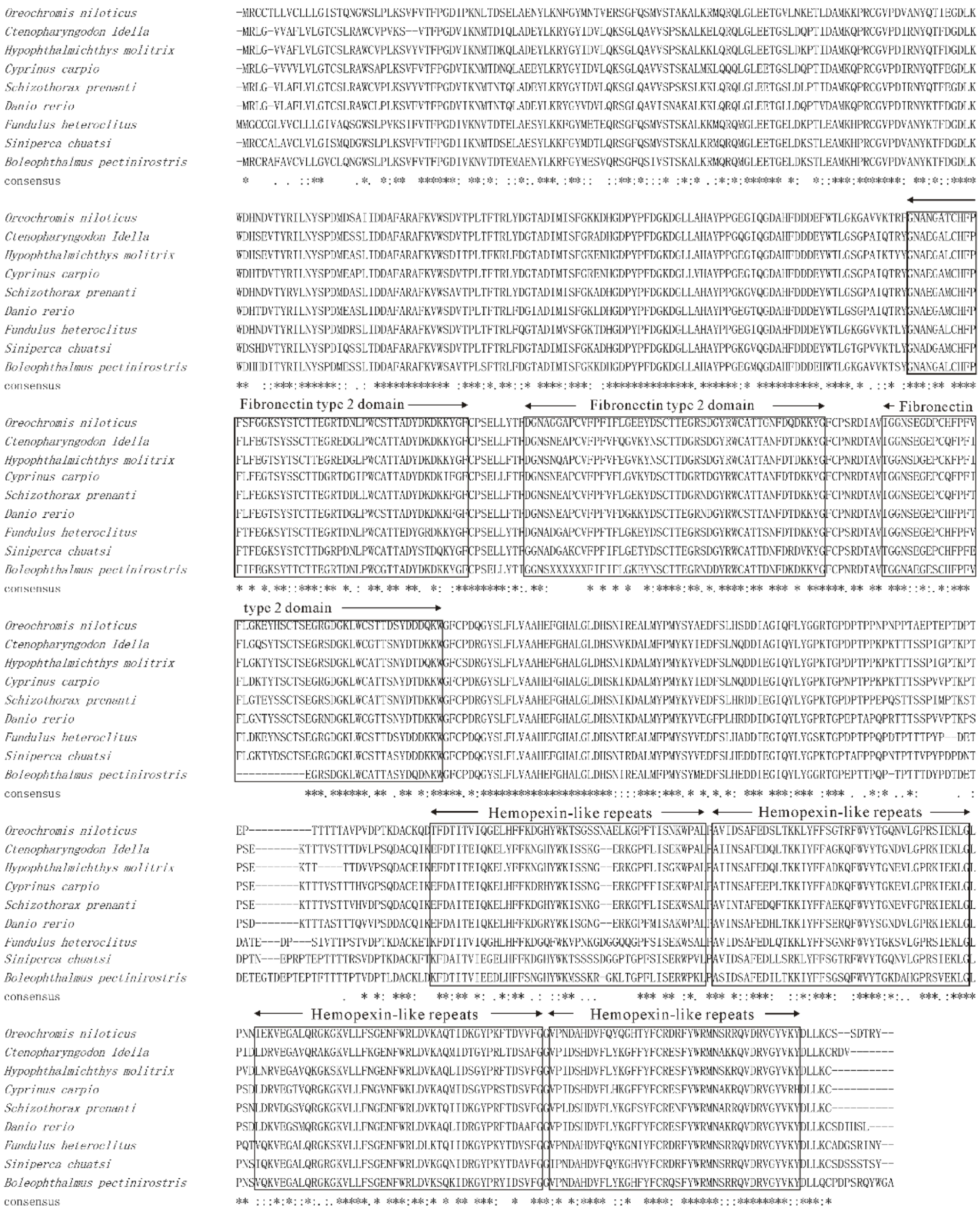


**Figure S3.** Multiple amino acid sequences alignment of MMP-9 from Nile tilapia and other species.

Amino acid sequences of MMP-9s from other species, including *Ctenopharyngodon idella* (Accession No. ADU34085.1), *Hypophthalmichthys molitrix* (APO13601.1), *Cyprinus carpio* (BAB39390.1), *Schizothorax prenanti* (AMB72630.1), *Danio rerio* (AAI60656.1), *Fundulus heteroclitus* (XP_012726094.1), *Siniperca chuatsi* (AKA66298.1), and *Boleophthalmus pectinirostris* (XP_020785344.1).

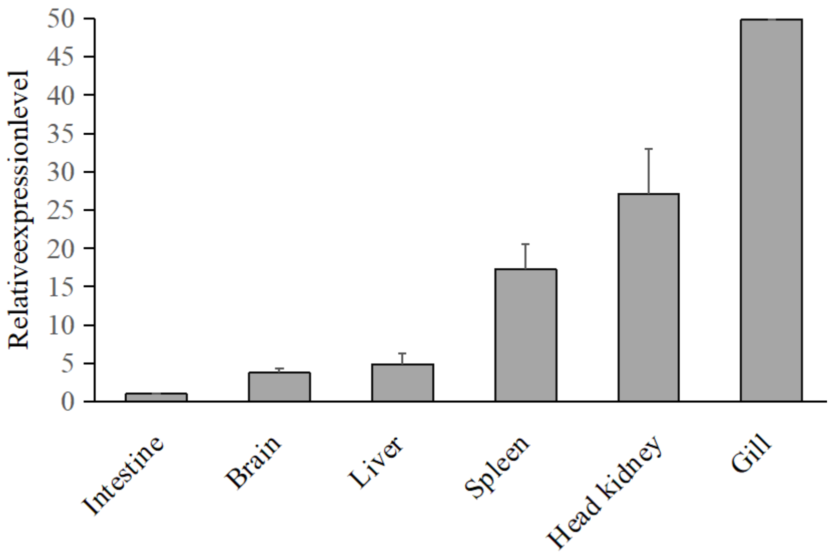


**Figure S4.** Relative transcriptional levels of *NtMMP-9* in different tissues from healthy fish. The transcriptional value of *NtMMP-9* in the intestine was used as the internal reference.

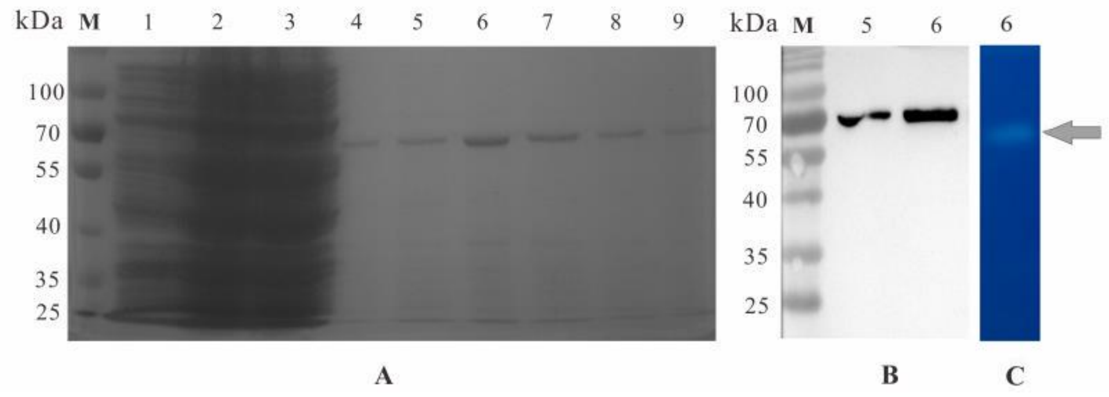


**Figure S5.** Electrophoretic analysis of recombinant aNtMMP-9 expressed from Rosetta-gami 2 (DE3) competent cell.

(A) SDS-PAGE of aNtMMP-9 expressed by Rosetta-gami (DE3) at 16 °C. M, protein maker; Lane 1, the total protein expressed without IPTG; Lane 2, the total protein expressed after IPTG (0.5 mM) induction for 12 h; Lane 3, unbound protein; Lane 4, the protein from last washing with imidazole (200 mM); and Lanes 5–9, the proteins collected using an elution buffer (0.5 mL, 200 mM imidazole) and repeated five times. (B) Western-Blotting analysis for the proteins obtained from lanes 5 and 6 in Figure S4A. (C) Gelatin zymography of the protein harvested from that of Figure S4B. The molecular weight (63.4 kDa) of the obtained aNtMMP-9 was denoted by an arrow.

# Supplementary tables

**Table S1.** Information of experimental fish used in this study

| Fish | Fish number per tank | Tank number | Fish number in different time points after *S. agalactiae* challenge for collecting their tissues | | | |
|---|---|---|---|---|---|---|
| | | | 4 h | 24 h | 48 h | 72 h |
| Healthy group | 10 | 1 | Three healthy fish for collecting tissues and identifying the transcriptional levels of *NtMMP-9* gene | | | |
| Control group | 10 | 3 | 3 | 3 | 3 | 3 |
| Challenged group | 10 | 3 | 3 | 3 | 3 | 3 |

Notes: Eighty fish were purchased, and 76 of them were used in this study. Three fish was used to perform the pathogen-free detection. Three fish was used to extract the total RNA and synthesize cDNA. Others of the 76 fish were divided into three groups according to those described in Materials and Methods.

**Table S2.** Interactions between NtMMP-9 and other proteins in Nile tilapia under *S. agalactiae* infection.

| Abbreviation | Protein/Accession number | Abbreviation | Protein/Accession number |
| --- | --- | --- | --- |
| HB-EGF | Proheparin-binding EGF-like growth factor isoform X1 (XP_019200773.1) | MMP-14 | Matrix metalloproteinase-14 (XP_003457484.1) |
| CYBA | Cytochrome b-245 light chain (XP_003442297.1) | MMP-15 | Matrix metallopeptidase-15 (XP_003437629.1) |
| TIMP-2(1) | Metalloproteinase inhibitor 2 (XP_003450177.1) | DCN | Decorin precursor (NP_001266536.1) |
| TIMP-2(2) | Metalloproteinase inhibitor 2 (XP_003441786.1) | PTK | Proto-oncogene tyrosine-protein kinase Src isoform X4 (XP_003438972.1) |
| TIMP-4 | Metalloproteinase inhibitor 4 (XP_003454588.1) | TIMP-3 | Metalloproteinase inhibitor 3 (XP_005939292.1) |
| Collagenase 3 | Collagenase 3 (XP_013129643.2) | Thbs2 | Thrombospondin-2 isoform X3 (XP_005474097.1) |
| IGF-I | Insulin-like growth factor I (AAC17494.1) | Plat | Tissue-type plasminogen activator isoform X1 (XP_005473584.2) |
| ITGB4 | Integrin beta-4 isoform X2 (XP_013129071.1) | Plasminogen | Plasminogen (XP_005461555.1) |
| Thbs1b | Thrombospondin-1b isoform X1 (XP_005453322.1) | Lama4 | Laminin subunit alpha-4 (XP_005457978.1) |
| Thbs1a | Thrombospondin-1a precursor (NP_001266664.1) | LYVE1 | Lymphatic vessel endothelial hyaluronic acid receptor 1 isoform X4 (XP_005471040.1) |

Note: The data of Nile tilapia were from the STRING 10.5 (http://string-db.org/) of Nile tilapia.

**Table S3.** Putative binding sites of NtMMP-9 interacting with DCN

| Interaction | NtMMP-9 aa | DCN aa | Length (Å) |
|---|---|---|---|
| Hydrophobic interaction | Cys-376, Cys-561 | Trp-335 | |
| Hydrophobic interaction | Phe-538, Trp-540, Val-551, Gly-552, Tyr-553 | Leu-327, Phe-328, Val-332, Pro-333, Leu-353 | |
| Cation-π interaction | Lys-377 | Trp-335 | |
| Anion-π interaction | Asp-557 | Tyr-334 | |
| CH-π interaction | Phe-538, Trp-540 | Phe-328 | |
| Hydrogen bond | Lys-377 | Glu-336 | 3.1 |
| | Tyr-553 | Leu-327 | 2.3 |
| | Val-551 | Leu-353 | 2.4 |

**Table S4.** Putative binding sites of NtMMP-9 interacting with TIMP-2

| Interaction | NtMMP-9 aa | TIMP-2 aa | Length (Å) |
|---|---|---|---|
| Hydrophobic interaction | Phe-183, Phe-209, Trp-202 | Ala-197 | |
| Hydrophobic interaction | Phe-111, Phe-168, Gly-170, Ala-172, Tyr-200, Tyr-215, Phe-217 | Met-150, Val-194, Tyr-204, Val-207, Ala-208 | |
| Cation-π interaction | Tyr-215 | Arg-196 | |
| CH-π interaction | Tyr-215 | Tyr-204 | |
| Hydrogen bond | Asp-39 | Gln-84 | 2.7 |
| | Ala-31 | Thr-83 | 2.5 |
| | Tyr-12 | Gly-81 | 3.2 |
| | Tyr-49 | Gln-149 | 3.4 |
| | Ser-23 | Gln-146 | 3.5 |
| | Arg-196 | Met-150 | 2.8 |
